# A High Performance Wheat Disease Detection Based on Position Information

**DOI:** 10.3390/plants12051191

**Published:** 2023-03-06

**Authors:** Siyu Cheng, Haolan Cheng, Ruining Yang, Junyu Zhou, Zongrui Li, Binqin Shi, Marshall Lee, Qin Ma

**Affiliations:** 1 Yantai Institute of China Agricultural University, China Agricultural University, Yantai 264670, China; 2 International College Beijing, China Agricultural University, Beijing 100083, China; 3 College of Economics and Management, China Agricultural University, Beijing 100083, China; 4 College of Information and Electrical Engineering, China Agricultural University, Beijing 100083, China; 5 College of Plant Protection, China Agricultural University, Beijing 100083, China

**Keywords:** position attention, deep learning, machine learning, position-aware

## Abstract

Protecting wheat yield is a top priority in agricultural production, and one of the important measures to preserve yield is the control of wheat diseases. With the maturity of computer vision technology, more possibilities have been provided to achieve plant disease detection. In this study, we propose the position attention block, which can effectively extract the position information from the feature map and construct the attention map to improve the feature extraction ability of the model for the region of interest. For training, we use transfer learning to improve the training speed of the model. In the experiment, ResNet built on positional attention blocks achieves 96.4% accuracy, which is much higher compared to other comparable models. Afterward, we optimized the undesirable detection class and validated its generalization performance on an open-source dataset.

## 1. Introduction

Wheat is the second largest crop in the world, providing 19% of human caloric intake [1]. Wheat diseases greatly affect wheat production and cause significant wheat losses. At the current level of plant protection technology, annual wheat losses due to wheat diseases account for 26–30% of the theoretical wheat yield worldwide. In the absence of the application of plant protection technologies to manage farmland, wheat disease losses can account for up to 70% of the theoretical wheat yield [2]. Given that wheat leaves suffering from different kinds of diseases have certain differences, this is well suited to identify such differences and thus give diagnostic conclusions through computer vision-related methods. Nema et al. divided the collected images of wheat leaves into training and test datasets and tried to classify wheat leaf images by support vector machines to achieve differentiation between healthy wheat leaves and diseased wheat leaves [3]. Zhang et al. used the least squares discriminant support vector machines, K-nearest neighbor model and analysis model, and designed multiple sets of experiments to identify wheat grains with or without Fusarium spike blight. They chose hyperspectral images as training data, and the support vector machines achieved the best results on two images that were not used as training data [4].

In recent years, there have been studies on the application of computer vision techniques to the detection of wheat diseases. However, their training and test datasets are relatively small and the types of diseases that can be identified are single, and many of them research the issue through the method based on the support vector machine model. The drawback of support vector machines is that they require manual extraction of features from images. With the development of artificial neural networks, convolutional neural networks are becoming more and more mature. Using convolutional neural network techniques can avoid manual extraction of image features, which can reduce the burden of the researchers involved, which can make more researchers focus more on improving the accuracy of the model [5]. Convolutional neural networks are composed of many computational nodes and the nodes are arranged in the layer order. Each node in the previous layer generates data that is processed by an activation function and passed to each node in the next layer. When dealing with computer vision problems, images are often stored in RGB format. RGB images have three color channels and use a three-dimensional matrix to portray the image features [6]. Feature extraction is achieved through filters by acting on the matrix of features to be extracted. The presence of an activation function makes the 3D matrix of the original image and the matrix obtained by filter extraction, not simply linear [7]. By processing the images, the convolutional neural network can effectively perform the classification task. For wheat disease identification, data from the training set are input into the model, while the model weights are updated by backward transfer, and the predicted and expected values are used to calculate the error. After a limited number of epochs, the model and its parameters are saved, and the trained model can then be used to classify and detect a wide range of diseases.

Convolutional neural networks (CNN) have been applied to detect serval diseases in different plants [8,9,10,11,12,13,14,15,16], Marco Javier Suarez Baron, Angie Lizeth Gomez, and Jorge Enrique Espindola Diaz used CNN for image classification to detect late blight in potatoes, using a large number of images preprocessed to more easily extract features of late blight as an initial data set, and then using a classification model to identify whether the potatoes were infected or not. After that, an additional data set was set up for validation, image classification, and late blight detection. CNN has reached a maximum accuracy of 93.2% in identifying late blight diseases [17]. Ying Li, Shiyu Sun, Changshe Zhang, Guangsong Yang, and Qiubo Ye combined CNN with other multiple technologies for maize leaf disease detection. They added a CA attention module with key feature weights to improve the effectiveness of the feature map and an SSP module to reduce the loss of feature information based on a single-level plant disease network YoLov5s. They conducted three experiments using the new combined CNN model under conditions of overlapping occlusion, sparse distribution of detection targets, the texture of disease regions and similar backgrounds. This MFF-CNN has a higher average accuracy and shorter detection time compared to other methods like YoLov5s, Faster RCNN, CenterNet and DETR [18]. Xiaojuan Liu, Shangbo Zhou, Shanxiong Chen, Zelin Yi, Hongyu Pan, and Rui Yao used CNN image processing techniques to automatically identify buckwheat diseases using cosine similarity-based convolution and adding a two-level perceptual structure. The accuracy, recall, and F1 values of the updated CNN for disease detection were 97.54%, 96.38%, and 97.82%, respectively [19].

Lingwal et al collected 15,000 photographs of 15 wheat grains and successfully achieved the classification of wheat grains with 95% accuracy [20]. Using more than 12,000 photographs of wheat ears with wheat grains, Goyal et al. trained the resulting model to distinguish 10 wheat diseases with accuracy and recall of more than 95% [21]. The above studies suggest that the classification accuracy of the model can be effectively improved by using a large sample of the dataset.

It is noticeable that the dataset used by the above researchers consists of simple images of wheat ears or wheat leaves, and the model trained by such data has high requirements on the dataset, such as shooting angle, lighting conditions, and image background, and is therefore not suitable for deployment in real agricultural scenarios. Since the research in the field of real-time recognition of multiple diseases by inputting images taken in the wheat field with complex background is worth to be improved, this study proposes a fast and efficient wheat disease detection model based on a convolutional neural network model with a large sample training set that can be deployed in mobile scenarios based on lightweight technology for agricultural complex backgrounds.

The dataset used in this paper is photographed from diseased wheat grown in the field, which has a more complex picture background than previous studies with high image resolution and many pixel points. This adds to the complexity of the features extracted by the neural networks in our study. Moreover, convolutional neural networks are characterized by a large number of parameters, which leads to a long inference time and a large model size. The limited memory of mobile devices affects the operation of larger-size models and has an impact on the widespread use of models. Therefore, a lightweight design of the model is indispensable to enable the deployment of real-time detection on mobile. Driven by these deficiencies, the main novelty of this work is:

1. To solve the problem of lack of data sets, we propose a corresponding data augmentation method.
2. Based on feature map position information, a position attention block is proposed and implemented based on PyTorch.
3. In this paper, we conducted several experiments to verify the effectiveness of the position attention block and compared it with other attention blocks.

The rest of this paper consists of four sections. In Section 3, the design details of our model and the dataset are presented; Section 2 and Section 4 are presentation of our experimental results, for which we analyze and validate the effectiveness of our optimization method with a large number of experiments and analyze the limitations of our proposed method; in Section 5 we conclude the whole paper.

## 2. Results

### 2.1. Experiment Results

In this section, we experiment on AlexNet [22], VGG [23], MobileNet [24], ResNet [25], and GoogLeNet [26], and then add the position attention block proposed in this paper to the above five CNNs. The effectiveness of this method is demonstrated by comparing the effect of the model before and after the modification. The experimental results are shown in Table 1.

From the above table, we can see that the proposed position attention block can improve the model accuracy on many CNN models. On the ResNet model, it can improve the accuracy of the model by up to 2.7%. Although on GoogLeNet, PA does not improve the model effectively, which may be due to the complex parallel structure in GoogLeNet.

### 2.2. Validation on Other Datasets

To validate that the method proposed in this paper is effective and can be used for other agricultural tasks. The dataset used in this section, PlantDoc, was released by researchers at the Indian Institute of Technology (IIT), and it collects 27 categories (including 17 diseases such as bacterial disease of bell pepper, black rot of apple, powdery mildew of cherry, healthy disease of blueberry, the early blight of potato, the gray spot of corn, black rot of grape, etc. and 10 healthy plants) and 13 plant species, and the dataset contains 2567 images, which consisted of 2328 images from the training set and 239 images from the test set the resolution of each image in the dataset is 416 × 416. The experimental results are shown in Table 2.

From the above table, we can see that our method can still achieve 0.51 mAP on the PlantDoc dataset. This indicates that our method can also detect the disease well on other datasets, which shows that our method has good robustness.

### 2.3. Comparison with Other Attention Blocks

To verify the effectiveness of the position attention blocks, in this section, we compare other attention blocks based on ResNet and MobileNet. Specifically, we add the SE block, CBAM, and ECA block, which are the three attention blocks mentioned in Section 3.1, to ResNet and MobileNet in turn, and compare them with the position attention block. The dataset is a subset of this paper’s dataset, and the evaluation metric is accuracy. Experimental results are shown in Table 3.

From the above table, it can be seen that our method is optimal for improving the accuracy of the model in MobileNet and ResNet. Compared with the baseline, our method can effectively improve the accuracy by 2.1% and 2.2%, respectively. This result is 1.8% ahead of other attention blocks, which is a good demonstration of the effectiveness and efficiency of this method.

## 3. Materials and Methods

### 3.1. Backgounds

#### 3.1.1. Convolutional Neural Network (CNN)

CNN is a class of Feedforward neural networks with convolutional computation and deep structure, which is one of the representative algorithms of deep learning. CNN is capable of representation learning and shift-invariant classification of the input information according to their hierarchical structure. CNN is built to emulate the visual perception mechanism of living beings and can perform supervised and unsupervised learning with no additional feature engineering requirements on the data.

#### 3.1.2. Squeeze-Excitation (SE) Block

**Figure 1:**
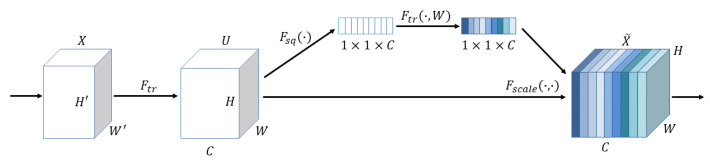
Illustration of squeeze-excitation block.

The SE block was first proposed in the paper [27], and its structure is shown in Figure 1.

The input *X* becomes the output *U* after an arbitrary transformation. Assume that the output *U* is not optimal and that each channel has a different degree of importance, with some channels being more useful and some less useful. Therefore, for each output channel, global average pooling is performed first, each channel gets a scalar, *C* channels get *C* numbers, and then after FC-ReLU-FC-Sigmoid gets *C* scalars between 0 and 1, as the channel weights. Finally, each channel of the original output channel is weighted with the corresponding weight (each element of the corresponding channel is multiplied with the weight separately), and the new weighted feature map is obtained, which the authors of the paper [27] call feature recalibration.

#### 3.1.3. Efficient Channel Attention (ECA) Block

**Figure 2:**
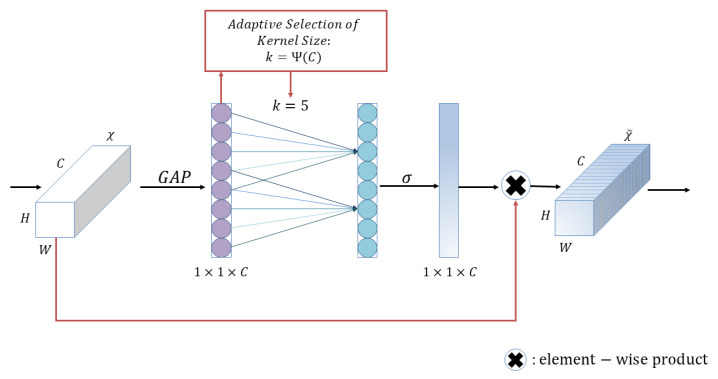
Illustration of efficient channel attention block.

ECA block, which is proposed in ECA-Net [28]. It is a channel attention block and is often applied to visual models. It supports plug-and-play, i.e., it can perform channel feature enhancement on the input feature map and output the final ECA block without changing the size of the input feature map. Its structure is shown in Figure 2.

First, input the feature map, which has dimension H×W×C; after that, compress the feature maps with spatial features; in the spatial dimension, use global average pooling to obtain a feature map with 1×1×C. After that, the compressed feature map is subjected to channel feature learning; the importance between different channels is learned through 1×1 convolution, and the output dimension is still 1×1×C at this time. Finally, the channel attention is combined, and the feature map with channel attention 1×1×C, the original input feature map H×W×C, is multiplied channel by channel, and the feature maps with channel attention are finally output.

#### 3.1.4. Convolutional Block Attention Module (CBAM)

**Figure 3:**
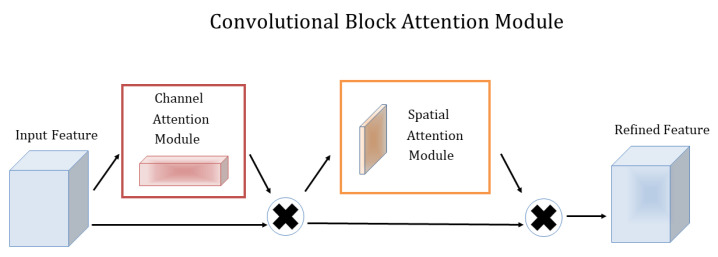
Illustration of convolutional block attention module.

The paper [29] proposes a lightweight attention module CBAM that can perform Attention in the channel and spatial dimensions. The paper adds the CBAM module to classical structures such as ResNet and MobileNet and performs a comparative analysis and visualization, and finds that CBAM is more focused on recognizing target objects, which also makes CBAM more interpretable, as shown in Figure 3.

CBAM consists of two separate sub-modules, the Channel Attention Module (CAM) and the Spatial Attention Module (SAM), CAM is in charge of performing channels and SAM is responsible for performing spatial Attention. This saves parameters and computing power while ensuring that it can be integrated into existing network architectures as a plug-and-play module. Attention on channels was proposed in SENet in 2017 [27], and CAM has only one more parallel max pooling layer compared to SENet. The paper also gives an explanation and experimental data support as to why this is changed.

##### Channel Attention Module

**Figure 4:**
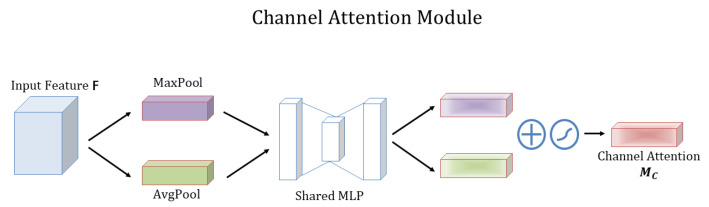
Illustration of channel attention module in CBAM.

The channel attention module and its computation are shown in Figure 4.

F(H×W×C) is the input feature map, and global maximum pooling and global average pooling are performed based on width and height to obtain two 1×1×C feature maps, which are fed into a two-layer neural network (MLP) and then activated by $\frac{C}{r}$ (*r* is the reduction rate). The result of $\frac{C}{r}$ (*r* is the reduction rate) represents the number of neurons in the first layer, the activation function is ReLU, and *C* represents the number of neurons in the second layer. Then, the output features of MLP are subjected to element summation operation and then sigmoid activation operation to produce the final channel attention feature, $M_{c}$. Finally, the element-wise multiplication operation is performed on $M_{c}$ and the input feature map *F* to produce the input features needed by the Spatial attention module.

##### Spatial Attention Module

**Figure 5:**
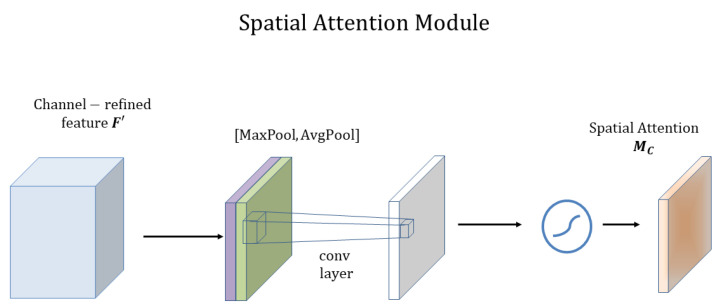
Illustration of spatial attention module in CBAM.

The spatial attention module and the specific computation are shown in Figure 5.

This module uses the feature map $F^{\prime }$ output from the channel focus module for the input feature map. First, do a channel-based global maximum pool and global average pool and get two H×W×1 feature maps, and then do a join operation (i.e., channel stitching) on these two feature maps based on channels. Then after based on the convolution of 7×7 (7×7 is better than 3×3), i.e., H×W×1, the dimensionality is reduced to 1 channel. Then, the spatial attention feature $M_{s}$ is generated by the sigmoid. Finally, this feature is multiplied with the input feature of this module to obtain the final generated feature maps.

### 3.2. Data Collection

**Figure 6:**
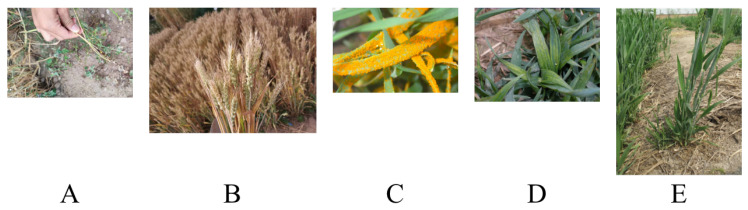
Examples of our dataset. (**A**) is the Macrophthalmia, (**B**) is the healthy, (**C**) is the rust, (**D**,**E**) are Blastomycosis.

Wheat was photographed during 2020 and 2022. Examples of images can be seen in Figure 6.

Canon EOS 5D Mark III Digital SLR Camera is used to capture all photographs which are from the Science Park of the West Campus of China Agricultural University, Benetwork g, China. The primary method was to capture images of a single image presenting symptoms of the disease while walking through rows of planted fields. The photographed wheat was simply found by chance in an area known for the disease. We attempted to photograph wheat at various heights in proportion to the distribution of disease symptoms. An amount of 10–20 images would be photographed after each walk ended.

We only photographed wheat diseases that naturally occurred in the field. 1337 images were captured by us in 2020. 1289 original images were captured by us during the 2021 season. All images were taken at the Science Park of the West Campus of China Agricultural University. The process of data acquisition we used was slightly modified based on the process we had used the year prior. The same Digital SLR camera was used for about 70% of cases, an iPhone was used for the other 30%. The purposes of doing this are better approaching the types of images that the application’s users might take in the field, and adding variety to the dataset which might be helpful for the model to distinguish between signal and noise. Increasing the proportion of images of wheat on distinct backgrounds was another modification. Single detached images took up about 30% in 2021. We photographed about half of the detached photographs with the wheat laid flat on the ground. Light conditions were also deliberately varied, we blended the subjects nearly evenly in both sunny and shady locations.

In total, we have collected 2626 original images across the seasons. To these original images, we added some additional images from extended sources and the Internet. The distribution of our dataset can be seen in Table 4.

### 3.3. Data Augmentation

The recognition of pests and diseases requires high environmental conditions (e.g., lighting and angles) for shooting, and the recognition accuracy is affected by the image quality. Therefore, we use data enhancement techniques to enhance the generalization ability of the model by randomly increasing or decreasing the brightness, randomly rotating and mirror flipping the existing images. Among them, when randomly increasing or decreasing the luminance, the following equation is used:

(1) Output=α×Input+β

**Figure 7:**
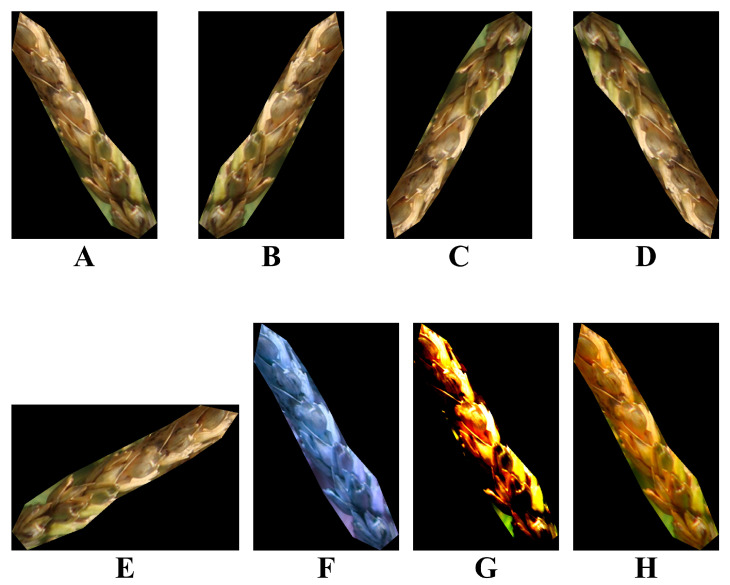
Illustration of different enhancement methods. (**A**) Original image. (**B**) Vertical flipping. (**C**) Horizontal flipping. (**D**) Horizontal and Vertical flipping. (**E**) Rotation of 90 degrees. (**F**) Hue adjustment. (**G**) Contrast adjustment. (**H**) Saturation adjustment.

In Equation (1) α stands for contrast, and β stands for brightness. After randomly increasing or decreasing the image luminance, the image is normalized to between [−1,1], and then the rotation center point is randomly rotated by a certain angle and then mirrored and flipped. All the enhancement operations are performed automatically during training, and the effects of various enhancement methods are shown in Figure 7.

### 3.4. Proposed Method

**Figure 8:**
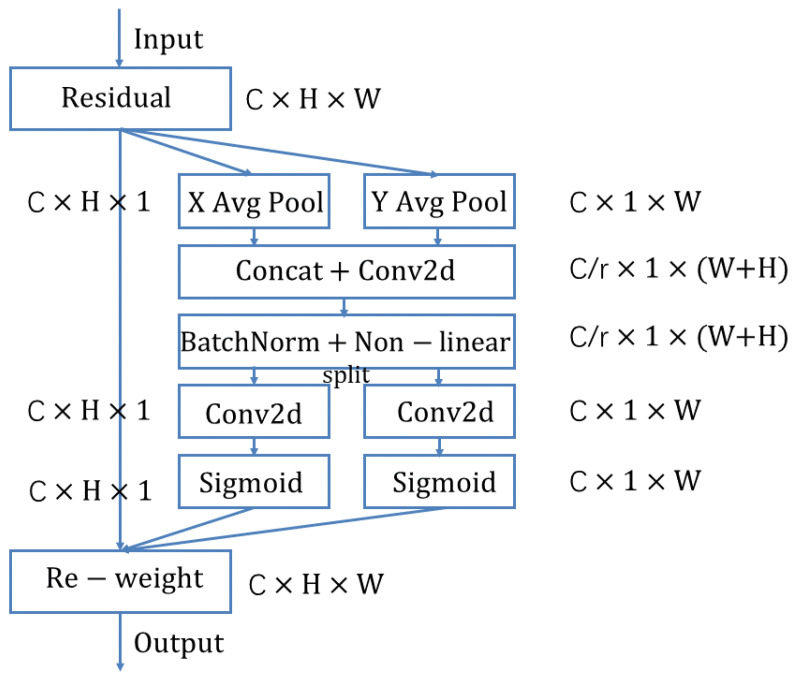
Illustration of position attention block.

The discussion in Section 3.1 shows that while channel attention improves model performance, it typically ignores position information that is important for generating spatially selective attention maps. Therefore, in this paper, we propose a novel attention mechanism specifically designed for lightweight networks, which embeds position information into channel attention. Unlike channel attention, which transforms the input into a single feature vector through two-dimensional global pooling, the approach we adopt decomposes channel attention into two one-dimensional feature encoding processes that cover features along different directions. This approach has the advantage of capturing the long-range along one direction while being able to maintain accurate position information along the other spatial direction and then encoding the generated feature maps separately to form a pair of position-sensitive feature maps that can be applied complementarily to the input feature maps to enhance the representation of the region of interest. The structure of the proposed method is shown in Figure 8.

#### 3.4.1. Position Attention Overview

Position attention uses two one-dimensional global pooling operations to aggregate the input features in the vertical and horizontal directions into two separate position-aware feature maps. These two feature maps embedded with direction-specific information are each encoded as two attention maps, where each attention map captures the long-range dependence of the input feature map along the spatial direction. Thus, the position information is stored in the generated attention graph and the two attention graphs are then multiplied onto the input feature graph to enhance the feature graph representation. Since this attention operation distinguishes spatial locations and generates feature maps based on the position information, the proposed method is referred to as position attention.

Compared with previous atdecomposethods on lightweight networks, our method has the following advantages:

1. It is flexible and lightweight, making it easy to plug into existing networks such as ResNet [25], VGG [23], and MobileNet [24], and to improve functionality by enhancing information representation.
2. It captures cross-channel information as well as position-aware information, which helps the model to more accurately locate and identify targets of interest.
3. Finally, position attention as a pre-trained model can bring significant benefits for downstream tasks on top of lightweight networks, especially those where intensive prediction exists, such as semantic segmentation, as discussed in Section 4.1.

#### 3.4.2. Position Attention Block

Position Attention Block encodes channel relations and long-range dependencies with accurate position information, similar to the SE block [27], and is divided into two steps: position information embedding and position attention generation, which is structured as follows. Global pooling is used to globally encode spatial information in channel attention and as channel descriptors, so this makes it difficult to retain position information. Therefore, this paper decomposes the global pooling into a pair of one-dimensional feature encoding operations to facilitate attention blocks to capture spatial long-range dependencies with precise positions. Specifically, for input *X*, each channel is first encoded along both horizontal and vertical directions using pooling kernels of dimensions H×1 and 1×W, so that the output of the first *c* channel with height *h* and the first *c* channel with width *w* are shown in Equation (2) and Equation (3), respectively.

(2) $$z_{c}^{h}\left(h\right)=\frac{1}{W}\sum_{0\leq i<W}x_{c}\left(h,i\right)$$

(3) $$z_{c}^{w}\left(h\right)=\frac{1}{H}\sum_{0\leq j<H}x_{c}\left(j,w\right)$$

To obtain a position-aware attention graph, the above two transformations perform feature aggregation on the position information, which is different from the way the SE block generates feature vectors in full. Also, these two transformations allow the attention module to capture long-distance dependencies along one spatial direction and retain precise position information along the other spatial direction, which helps the network to localize the region of interest more precisely.

### 3.5. Implement and Experiment

#### 3.5.1. Implement

We implemented a prototype of our methods in 3289 lines of Python and Java. We implemented a position attention block using PyTorch. We implemented the main construction and verification operations using the NumPy and sklearn Python library. We implemented and tested our method on various hardware platforms, including Linux desktops with GPUs, Apple laptops (M1 Max), and an NVIDIA Jetson Nano.

#### 3.5.2. Experiment Metric

**Figure 9:**
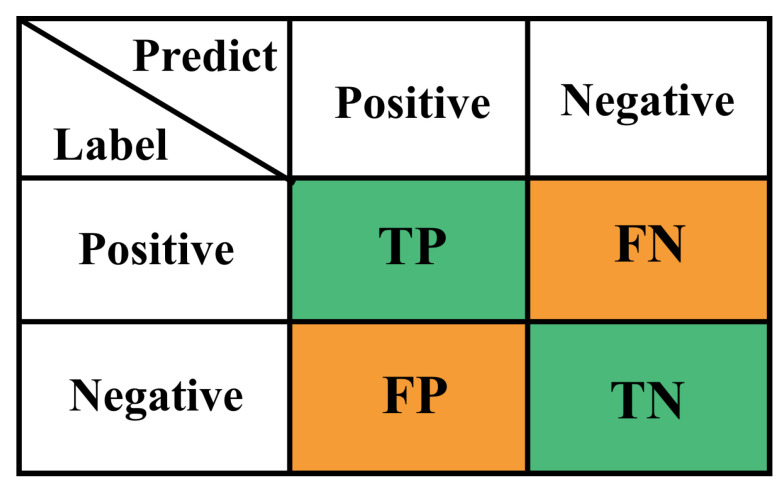
Confusion matrix with binary classification task.

When evaluating a multi-classification task, the confusion matrix is often used, with the true label on the left and the predicted label on the bottom. Take the binary classification task as an example, the confusion matrix is shown in Figure 9.

In this case, there are four cases:

1. Classifies a positive case as positive, which is denoted as true positive (*TP*).
2. Classifies a positive case as a negative case, which is denoted as a false negative (*FN*).
3. Classifies a negative case as negative correctly, denoted as true negative (*TN*).
4. Classifies a negative case as a positive case, denoted as false positive (*FP*).

The accuracy is calculated as follows:

(4) $$Accuracy=\frac{TP+TN}{N}$$

In Equation (4), *N* represents the number of samples tested, and TP+TN denotes the total number of correctly predicted samples in the test.

**Figure 10:**
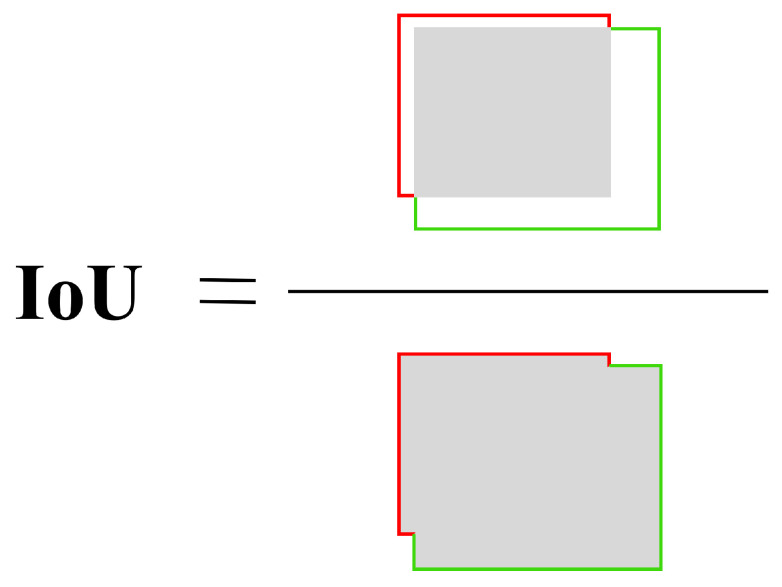
Illustration of the process of IoU calculation.

Further, the object detection task usually involves multi-class detection, where each image may contain several different classes of objects. Mean average precision (mAP) is a measure of the performance of each ground truth for each class. The ground truth involves the IoU evaluation metric, which is the ratio of the intersection and union sets of the prediction framework and ground truth. This quantity is also known as Jaccard’s index and was first introduced by Paul Jaccard in the early 20th century. To obtain the intersection and the union, we first put the prediction frame and the ground truth together. For each class, the region where the prediction frame and the ground truth overlap is the intersection, and the total region across is the union. Then IoU can be calculated as shown in Figure 10.

## 4. Discussion

### 4.1. Validation of Generality

To verify the generality of this method to other computer vision tasks, the backbone constructed based on the position attention block is put into the object detection network and semantic segmentation network for testing and the experimental results are shown in Table 5. The dataset used in this section is from the Science Park of the West Campus of China Agricultural University and the Internet.

**Figure 11:**
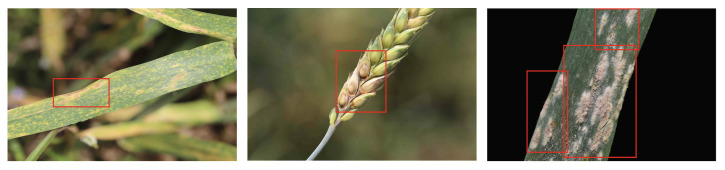
Illustration of object detection results. The red box indicates the location of the lesion.

From the above table, we can see that our method is still effective in object detection and semantic segmentation tasks. After adding the position attention block, the performance of the model is improved compared to the baseline. In the object detection task, since the objects to be detected are small-scale, as shown in Figure 11, the proposed attention mechanism can effectively improve the backbone’s ability to extract the region of interest, so the model performance is improved by 0.03 on YOLOv3.

### 4.2. Data Balancing

The dataset used in this paper contains 2626 images, including 1489 images of healthy wheat, as shown in Table 4. It can be seen that the dataset is not balanced. This imbalance is why the dataset is enhanced by the dataset enhancement method in this paper. In this section, we compare the performance of the model before and after the enhancement. Without the augmentation, the accuracy is only 91.7%, which is 4.7% lower compared to the augmentation. From this experimental result, we can see that balancing the dataset with data augmentation is important to improve the model performance.

## 5. Conclusions

In agricultural production life, the protection of wheat yield is a top priority. Controlling wheat diseases is one of the important initiatives to protect wheat yield effectively protects wheat yield, therefore, disease identification of wheat is extremely critical to promote agricultural development. In recent years, with the continuous development and innovation of computer vision technology, the implementation of various plant disease detection has become more solvable.

Deep learning has been commonly used in smart agriculture scenarios, and its models have the following characteristics: (1) Deep network models are very dependent on the dataset. (2) The performance of the deep model can be improved by the attention mechanism. (3) Most of the existing research focuses on recognition efficiency without paying attention to inference efficiency, which makes its application in practical production limited.

Therefore, the dataset used in this paper is photographed from diseased wheat grown in the field, which has a more complex picture background than previous studies with high image resolution and many pixel points. This adds to the complexity of the features extracted by the neural networks in our study. Moreover, convolutional neural networks are characterized by a large number of parameters, which leads to a long inference time and a large model size. The limited memory of mobile devices affects the operation of larger-size models and has an impact on the widespread use of models. Therefore, a lightweight design of the model is indispensable to enable the deployment of real-time detection on mobile. Driven by these deficiencies, the main novelty of this work is:

1. To solve the problem of lack of dataset, we propose a corresponding data augmentation method.
2. Based on feature map position information, a position attention block is proposed and implemented based on PyTorch.
3. In this paper, we conducted several experiments to verify the effectiveness of the position attention block and compared it with other attention blocks.

## Figures and Tables

**Table 1:** Results on our datasets via different CNNs.

Model	Input Size	Mean Accuracy (in %)
AlexNet	224 × 224	81.3 ± 1.7
AlexNet + PA	224 × 224	83.4 ± 0.7
VGG	224 × 224	85.6 ± 1.2
VGG + PA	224 × 224	86.9 ± 1.2
MobileNet	224 × 224	88.5 ± 2.0
MobileNet + PA	224 × 224	90.1 ± 1.4
ResNet	224 × 224	93.7 ± 0.8
ResNet + PA	224 × 224	96.4 ± 0.8
GoogLeNet	299 × 299	89.6 ± 0.9
GoogLeNet + PA	299 × 299	89.5 ± 0.8

**Table 2:** Results on other datasets via a backbone based on position attention block.

Model	Input Size	Pretrained Weights	mAP
MobileNet	416 × 416	COCO	0.42 ± 0.08
ResNet	416 × 416	COCO + PVD	0.51 ± 0.03

**Table 3:** Comparison with other attention blocks via MobileNet and ResNet (in %).

Backbone	Baseline	+SE	+CBAM	+ECA	+PA
MobileNet	92.3 ± 1.3	93.5 ± 1.3	93.6 ± 1.3	93.3 ± 1.2	94.4 ± 1.2
ResNet	93.8 ± 0.4	94.7 ± 0.4	94.7 ± 0.4	94.2 ± 0.4	96.0 ± 0.4

**Table 4:** Distribution of the wheat dataset in this paper.

Class	Number
Healthy	1489
Rust	378
Blastomycosis	296
Macrophthalmia	463

**Table 5:** Validation of position attention block’s generality based on object detection and semantic segmentation tasks.

Task	Model	Baseline	Ours
Object Detection	YOLOv3	0.84 ± 0.04	0.87 ± 0.04
	YOLOv5	0.87 ± 0.04	0.88 ± 0.03
Semantic Segmentation	MaskRCNN	0.72 ± 0.08	0.73 ± 0.07

## References

[1] Atchison J., Head L., Gates A. (2010). Wheat as food, wheat as industrial substance; comparative geographies of transformation and mobility. Geoforum.

[2] Mesterházy Á., Oláh J., Popp J. (2020). Losses in the grain supply chain: Causes and solutions. Sustainability.

[3] Nema S., Dixit A. Wheat leaf detection and prevention using support vector machine. Proceedings of the 2018 International Conference on Circuits and Systems in Digital Enterprise Technology (ICCSDET).

[4] Zhang L., Ji H. (2019). Identification of wheat grain in different states based on hyperspectral imaging technology. Spectrosc. Lett..

[5] Sakib S., Ahmed N., Kabir A.J., Ahmed H. (2018). An overview of convolutional neural network: Its architecture and applications. Preprints.

[6] Liu G.R. Rice color inspection based on image processing technique. Proceedings of the 2010 International Conference on Advances in Energy Engineering.

[7] Kiliçarslan S., Celik M. (2021). RSigELU: A nonlinear activation function for deep neural networks. Expert Syst. Appl..

[8] Zhang Y., Wa S., Liu Y., Zhou X., Sun P., Ma Q. (2021). High-Accuracy Detection of Maize Leaf Diseases CNN Based on Multi-Pathway Activation Function Module. Remote Sens..

[9] Wang L., Chen A., Zhang Y., Wang X., Zhang Y., Shen Q., Xue Y. (2020). AK-DL: A Shallow Neural Network Model for Diagnosing Actinic Keratosis with Better Performance than Deep Neural Networks. Diagnostics.

[10] Zhang Y., Liu X., Wa S., Liu Y., Kang J., Lv C. (2021). GenU-Net++: An Automatic Intracranial Brain Tumors Segmentation Algorithm on 3D Image Series with High Performance. Symmetry.

[11] Zhang Y., Wa S., Sun P., Wang Y. (2021). Pear Defect Detection Method Based on ResNet and DCGAN. Information.

[12] Zhang Y., He S., Wa S., Zong Z., Liu Y. (2021). Using Generative Module and Pruning Inference for the Fast and Accurate Detection of Apple Flower in Natural Environments. Information.

[13] Zhang Y., Liu X., Wa S., Chen S., Ma Q. (2022). GANsformer: A Detection Network for Aerial Images with High Performance Combining Convolutional Network and Transformer. Remote Sens..

[14] Zhang Y., Li M., Ma X., Wu X., Wang Y. (2022). High-Precision Wheat Head Detection Model Based on One-Stage Network and GAN Model. Front. Plant Sci..

[15] Zhang Y., Wa S., Zhang L., Lv C. (2022). Automatic Plant Disease Detection Based on Tranvolution Detection Network With GAN Modules Using Leaf Images. Front. Plant Sci..

[16] Zhang Y., Wang H., Xu R., Yang X., Wang Y., Liu Y. (2022). High-Precision Seedling Detection Model Based on Multi-Activation Layer and Depth-Separable Convolution Using Images Acquired by Drones. Drones.

[17] Suarez Baron M.J., Gomez A.L., Diaz J.E.E. (2022). Supervised Learning-Based Image Classification for the Detection of Late Blight in Potato Crops. Appl. Sci..

[18] Li Y., Sun S., Zhang C., Yang G., Ye Q. (2022). One-stage disease detection method for maize leaf based on multi-scale feature fusion. Appl. Sci..

[19] Liu X., Zhou S., Chen S., Yi Z., Pan H., Yao R. (2022). Buckwheat Disease Recognition Based on Convolution Neural Network. Appl. Sci..

[20] Lingwal S., Bhatia K.K., Tomer M.S. (2021). Image-based wheat grain classification using convolutional neural network. Multimed. Tools Appl..

[21] Goyal L., Sharma C.M., Singh A., Singh P.K. (2021). Leaf and spike wheat disease detection and classification using an improved deep convolutional architecture. Inform. Med. Unlocked.

[22] Krizhevsky A., Sutskever I., Hinton G.E. (2012). Imagenet classification with deep convolutional neural networks. Adv. Neural Inf. Process. Syst..

[23] Simonyan K., Zisserman A. (2014). Very deep convolutional networks for large-scale image recognition. arXiv.

[24] Howard A.G., Zhu M., Chen B., Kalenichenko D., Wang W., Weyand T., Andreetto M., Adam H. (2017). Mobilenets: Efficient convolutional neural networks for mobile vision applications. arXiv.

[25] He K., Zhang X., Ren S., Sun J. Deep residual learning for image recognition. Proceedings of the IEEE Conference on Computer Vision and Pattern Recognition.

[26] Szegedy C., Vanhoucke V., Ioffe S., Shlens J., Wojna Z. Rethinking the Inception Architecture for Computer Vision. Proceedings of the 2016 IEEE Conference on Computer Vision and Pattern Recognition (CVPR).

[27] Hu J., Shen L., Sun G. Squeeze-and-excitation networks. Proceedings of the IEEE Conference on Computer Vision and Pattern Recognition.

[28] Wang Q., Wu B., Zhu P., Li P., Zuo W., Hu Q. ECA-Net: Efficient channel attention for deep convolutional neural networks. Proceedings of the 2020 IEEE/CVF Conference on Computer Vision and Pattern Recognition.

[29] Woo S., Park J., Lee J.Y., Kweon I.S. Cbam: Convolutional block attention module. Proceedings of the European Conference on Computer Vision (ECCV).

